# Notes and News

**Published:** 1888-12-22

**Authors:** 


### Notes and News.

Collected and Communicated.

Next week we hope to publish some useful facts about deserving hospitals and charities for the guidance of those members of the public who desire to aid them with monetary gifts. Any facts showing the needs and claims of individual institutions should be addressed—The Editor, The Lodge, Porchester Square, W., and be posted not later than Monday.

Christmas entertainments are well to the fore now. At University Hospital Christmas is to be kept on December 31st, when invitations are issued for from 5 to 8.30. At the London Hospital the usual tree for the children will be on view in the Queen Wards at two o'clock on New Year's Day. At Great Ormond Street there is a carol service on December 22nd. In fact, invitations are just pouring in on us at this festive season.

Dr. Alice Marston has been doing good work at Lucknow lately for the Medical Branch of the Indian Female Normal School Society. During 1887 she had 110 in-patients, 5,000 out-patients, and 100 private patients. This society has five fully-qualified medical women on its staff, and hopes to soon open hospitals at Benares and Patna, where its missionaries are already at work. It costs £500 to train a lady doctor and send her to India.

The annual reports are coming in rapidly from Scotland. At Stirling the Royal Infirmary has benefited 202 in-patients, and 1,908 out-patients during the last year. Expenditure, £942; balance in hand, £31. The house-surgeon and matron both come in for warm commendation. At Ayr County Hospital there have been 485 in-patients and 1,562 out-patients during the last year. Expenditure, £1,753, leaving a debt of £13.

The Edinburgh Hospital and Dispensary for Women and Children flourishes under the medical superintendence of Dr. Sophia Jex-Blake. The expenditure during the last year has exceeded the income by £20 only, which is a very moderate amount of debt for a charitable institution. It is proposed to do away with debt altogether by teaching pupils of the School of Medicine for Women practical pharmacy. There have been 34 admissions of in-patients this year, and the out-patients have numbered 220.

The report just published of the Staffordshire Infirmary, tells how an epidemic of small-pox which threatened the town was promptly stamped out. A boy, admitted to the infirmary to be treated for lead poisoning, proved to have contracted small-pox before admission, and the disease was communicated to eight other patients in two wards, and also to a nurse and a ward maid. Prompt action was taken by the medical staff, the cases were at once removed to the infectious diseases hospital at Bucknall, the other patients of the infected wards were removed to a tent hospital specially erected in the grounds, and for a fortnight no fresh cases, except cases of accident and extreme urgency, were admitted to the infirmary. These means fortunately proved sufficient to stop the epidemic.

The cripples of Surrey can rejoice, for the miracle-working priest, Father Ignatius Larkin, has left Ireland for the South of England. Mr. Daly, Town Commissioner at Mullingar, gives particulars of some of Father Larkin's cures. A boy, aged seven, who had never been able to walk, found the use of his legs when prayed over by the Rev. Father; and Mr. Daly's own son, who had a stiff arm, was cured. Faith-healing is coming to the fore again evidently.

The Industrial Home for Crippled Boys in Wright's Lane, Kensington, holds 85 boys, who are kept for three years and taught a trade. An enlargement of the Home has lately been finished, but till a debt of £3,000 on it has been paid off the number of boys cannot be increased. It is indeed a good work which teaches even cripples to be independent, so that they need not be a burden to their friends or the community, and it is a work all may help, for orders for carpentering, tailoring, etc., are gladly received, and carefully executed by the afflicted inmates of the Home.

At the annual dinner of the Dental Hospital of London Mr. J. Smith Turner made a severe attack on the spurious dentists who infest the metropolis. The public are constantly being taken in by these cheap quacks, and it can only be said that they usually get a lot (of pain) for their money. Many who advertise themselves as practising American dentistry have never been within a thousand miles of America, and bring disgrace on the true professionals from that country. Mr. Smith Turner did not seem to consider that the title of L.D.S. was any criterion of worth.

A meeting of the Cardiff Infirmary Committee was held on December 12th, and the provision of additional accommodation was considered. The Chairman (Mr. Charles Thompson) expressed himself strongly in favour of adding a new block to the building, and offered to contribute £500 towards the cost. A resolution was passed thanking the chairman for his handsome offer, and the committee pledged themselves to use their best endeavours to raise the necessary funds. It is estimated that the cost of making the addition suggested would be about £4,000.

A successful meeting was held in the Lancaster Town Hall, on December 5th, to promote the building of a new infirmary for Lancaster. The trustees of the Ripley Hospital have presented a site, and subscriptions to the amount of £9,000 have been already collected. As the building is to cost not more than £15,000, it ought to be able to open free of debt. Mr. Henry Garnett has declared that the building shall have no architectural vagaries, but all money be employed for solid comfort. The workpeople and country districts are specially asked to come forward and help.

It must be rather a dreadful ordeal to propose the toast of "The Mile End Old Town Victoria Park Hospital Association"! We wonder the president of the Association, though he is Mr. Spencer Charrington, M.P., ever managed to get that long string of names out in their right order. But whatever the title, there is no doubt about the somewhat novel and thoroughly good work done by this society, of which the annual dinner was held last week. Managed by a committee largely composed of working men, this society collects small sums, from a penny a-week upwards, and invests them in life-governorships at the Victoria Park Hospital. During a very short time the association has bought 23 life-governorships, which are distributed by ballot, and which represent a sum of £724, paid into the funds of the hospital. This is the sort of charity which blesses both him that gives and him that takes; the working men are glad enough of the tickets which a governorship represents, and the hospital is glad enough of the £100 which it means to them.

The following vacancies are announced this week, the amount of salary in each case being given after the name of the institution :—

**Resident Medical Officers.**

General Hospital, Birmingham. Assistant House Surgeon — Board, etc.
Owen's College, Manchester. Junior Demonstrator in Zoology.— £100.
Ballymena Union. Medical Officer.—£120, fees, etc.

**Non-resident Medical Officers.**

Evelina Hospital, Southwark. Physician to Out-patients.
Birmingham Medical Aid Association. Medical Officers.
Leicester United Friendly Societies. Medical Officer.—£200, house, etc.
Hospital for Consumption, Brompton. Assistant Physician.

---

The Empress Frederick is untiring in her interest in medical charities. On Monday last she visited the Hospital for Sick Children, Great Ormond Street, and went the whole round of the wards, speaking to every little patient. A very small patient presented the Empress with a bouquet of violets. The Empress has also joined the Order of St. John of Jerusalem, and has accepted the badge of a Lady of Justice.

---

At the last meeting of the Metropolitan Asylums Board the clerk of the Battersea vestry called attention to the fact that measles was a dangerous infectious disease of a febrile character, and asked if the managers could not take measle cases into their hospitals. Dr. Felce said this was a question which ought to be faced. If the death-rate was 140 per week from smallpox in London there would be a great outcry, but because measles chiefly affected children, small notice was taken of it. There is some truth in Dr. Felce's remark.

---

Children have a bad time of it pretty often when they are too young to complain. This week it has almost seemed as though parents were anxious to get all their small offspring into the hospitals for Christmas. A baby two days old, wrapt in a thin shawl, was carried through Monday's fog to a hospital to be operated on for hare-lip. The friende were very indignant because the surgeon sent the child back to its mother. Another child, aged 21 hours, was taken to the priest to be baptised, and naturally died from the exposure. No wonder the death-rate from bronchitis is high.

---

The Vicar of St. Jude's, Southwark, has issued an appeal for funds to those who are not his parishioners. He says his own people do all they can, but so poor is the neighbourhood that the task of making necessary restoration of church and schools is beyond their means. From their vicar's amusing yet touching appeal, it would seem that the people of St. Jude's are a strange lot. The population (numbering nearly eight thousand) is composed of struggling artizans, small traders, makers of such curious commodities as dolls' eyes, small corks, tin toys, toothpicks, and pill boxes; and lastly, the unemployed or unemployable poor. Small tenements are gradually giving place to enormous blocks of model dwelling-houses, sheltering hundreds of families beneath one roof. The occupants of these human ant hills are for the most part of the migratory class, and to keep pastoral watch and ward over such is like attempting to herd a flock of wild sheep. There is a sensible ring about Mr. Pitchford's words, and those who appreciate a judicious combination of work and prayer, ought to heartily respond to his needs. "Religion apart," he says, "I can conceive no more sensible and humane organization for coping with the evils of our day than our National Church and Parochial System. But a system without agents and means to carry it into practice is like machinery without a motive power, and I fear nothing short of a definite and universal upholding and revivifying of our system will save it from the fate of any other organization which has become unable for its work."

An absurd fuss is being made about the supposed cruelty of innoculating Keanu with leprosy, and Dr. Arning is getting no credit for his devotion to science. Nothing too good can be said of Father Damien for living amongst, and ministsring to the lepers of Molokai. A draft for £1,000 has just been sent him, together with a letter of praise—almost of worship —from the Rev. Hugh Chapman, of Camberwell. Does it not occur to the public that Dr. Arning also lives amongst the lepers, and runs the risk of contagion? Father Damien is worthy of all the panegyrics he gets, but a little praise might be spared for Dr. Arning.

---

The ninth annual exhibition of home-made and other toys for distribution amongst the children in the various London hospitals, workhouses, workhouse schools, and infirmaries, was held in the Portman Rooms, Baker Street, on Thursday and Friday. There are over 22,000 children in these institutions, and it is intended to give each of them a separate toy for his or her own use, besides the large and more expensive toys which are presented to the various hospitals and workhouses for the general use of the inmates. There were some 23,000 to 24,000 toys on exhibition. A special feature of the show was the 3,858 dolls which have been dressed by lady readers of "Truth," in connexion with the competition for prizes for the best dressed doll.

---

An entertainment took place at the National Hospital for the Paralyzed and Epileptic, Queen Square, on December 6th, being the first of the winter series given fortnightly for the patients, many of whom were present on couches and invalid chairs, and showed their hearty appreciation of the efforts on their behalf. The instrumentalists were Miss Marianne Rock, professor of pianoforte at the Royal Albert Hall Orchestral Concerts, who gave with brilliant execution a gavotte; the violin was Miss C. Everett Green; the vocalists were Miss Louise Rock, who, in "Comin' thro' the Rye" and "Esmeralda," was much applauded and encored: Miss Mabel Hayward surpassed herself in "I know that my Redeemer liveth," and expressively sang in duets with Mr. G. Cosby Harpour, whose perfect rendering of "Comfort ye" and "Every Valley" was indeed worth hearing. "The King's Own" was sung by Mr. A. R. Walford, and encored. Readings were given by the Rev. J. A. Bailey and Mr. Rawlings. The chair was taken as usual by the Rev. E. H. Moran, chaplain. Another entertainment was given on Thursday last by the Resident Staff.

---

At the last meeting of the Metropolitan Asylums Board the following provisions for the Bill to be brought before Parliament with regard to infectious diseases were agreed upon :—(a) Legalise the admission into the managers' hospitals, upon certificates given by any qualified medical practitioner or medical officer of health, of cases of infectious disease, including diphtheria, of the non-pauper class, and authorise the managers to charge all expenses incurred in respect thereof direct to the respective vestries and district boards of the metropolis. (b) Confer upon the managers power to take compulsorily such sites and additional land and premises as shall be required for infectious hospital purposes; and (c) Compel the notification, by medical practitioners, of all cases of infectious disease which may be brought under their notice. Because of the urgent nature of the case, it was also agreed : —"That a communication be addressed to the President of the Local Government Board expressing the strong hope that the present session should not be allowed to expire without the passing of a measure for the purpose of relieving certain doubts as to the Board's dealing with the pauper sick; but if so, further expresses the earnest hope that the Board will take such steps in the ensuing session." There can be no doubt now that the notification of, and asylums for, infectious cases, will soon be put upon a proper legal footing.

